# Supplementary material for: T4SE-XGB: Interpretable Sequence-Based Prediction of Type IV Secreted Effectors Using eXtreme Gradient Boosting Algorithm
Source: Front Microbiol. 2020 Sep 24;11:580382. doi: 10.3389/fmicb.2020.580382 (PMC7541839; doi:10.3389/fmicb.2020.580382)
Supplement: Supplementary file 1 [file Table_1.DOCX]

***Supplementary Material***

**T4SE-XGB: interpretable sequence-based prediction of type IV secreted effectors using eXtreme gradient boosting algorithm**

Tianhang Chen^1^, Xiangeng Wang^1^, Yanyi Chu^1,2^, Yanjing Wang^1^, Mingming Jiang^1^ , Dong-Qing Wei^1,2,*^, and Yi Xiong^1, *^

^1^ State Key Laboratory of Microbial Metabolism, and School of Life Sciences and Biotechnology, Shanghai Jiao Tong University, Shanghai 200240, China

^2^ Peng Cheng Laboratory, Vanke Cloud City Phase I Building 8, Xili Street, Nanshan District, Shenzhen, Guangdong, 518055, China

* Correspondence:

Yi Xiong

[xiongyi@sjtu.edu.cn](mailto:xiongyi@sjtu.edu.cn)

Dong-Qing Wei

[dqwei@sjtu.edu.cn](mailto:dqwei@sjtu.edu.cn)

**Table S1.** Hyperparameters search range for eight different classifiers.

| **Method** | **Parameter** | **Tested values** |
| --- | --- | --- |
| RF and ERT | Number of estimators (n_estimators^[a]^) | 100–1000 with an interval of 100 |
|  | Maximum number of features considered per split (max_features^[a]^) | ['sqrt', 'log2'] |
| GB and XGB | Number of estimators (n_estimators^[a]^) | 100–1000 with an interval of 100 |
|  | Learning rate (learning_rate^[a]^) | [0.001,0.01,0.1,0.2,0.3] |
| ML | Number of neurons in the 1th hidden layer | [16,32,48,64] |
|  | Number of neurons in the 2th hidden layer | [16,32,48,64] |
| SVM | Penalty parameter (C^[a]^) | [2^-6^–2^6^] in log_2_ steps |
|  | Kernel coefficient (gamma ^[a]^) | [2^-6^–2^6^] in log_2_ steps |
| *k*-NN | Number of neighbors (n_neighbors^[a]^) | 1-41 with an interval of 1. |
| *LR* | Algorithm to use (solver^[a]^) | ['newton-cg','lbfgs','liblinear','sag'] |

Column 1 represents the ML algorithms. Column 2 and 3 represent the parameter name and the tested values, respectively. ^[a]^ Parameter name in the Scikit-learn implementation.

**Table S2.** Optimal parameter combination for each algorithm

| Method | Parameter |
| --- | --- |
| SVM | Gamma = 0.03125, C = 4 ^[a]^ |
| LR | multi_class ='auto', solver = 'liblinear' ^[a]^ |
| GB | n_estimators = 300, learning_rate = 0.2 ^[a]^ |
| XGB | n_estimators = 700, learning_rate = 0.1 ^[a]^ |
| RF | n_estimators = 300, max_features = 'sqrt' ^[a]^ |
| ERT | n_estimators = 900, max_features = 'sqrt' ^[a]^ |
| KNN | n_neighbors = 2 ^[a]^ |
| ML | hidden_layer_sizes = (48,16), max_iter = 1000 ^[a]^  all are default values |
| NB |  |

^[a]^ the values of all other parameters selected as default in the Scikit-learn implementation.

**
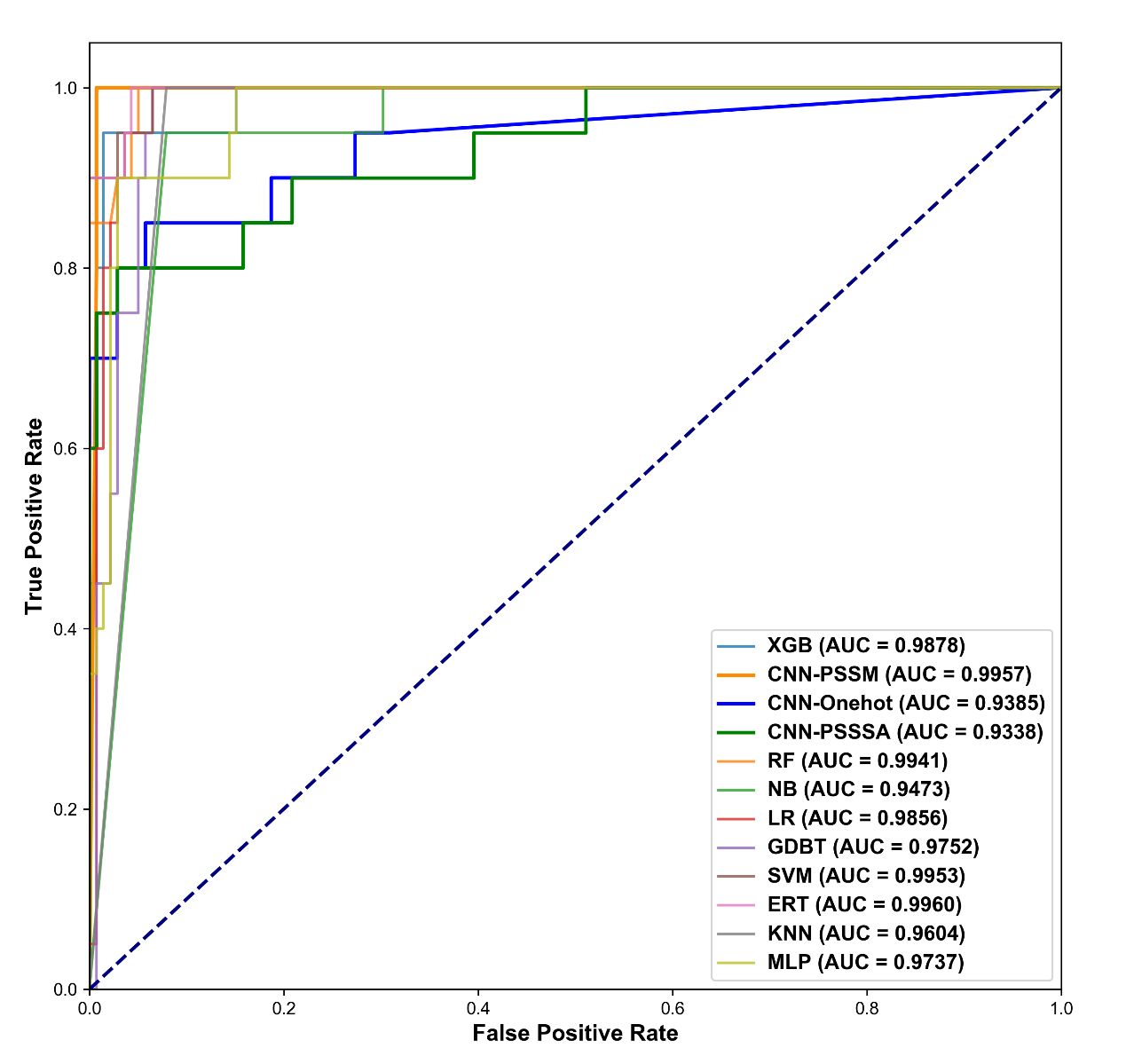
Figure S1.** ROC curves of different classification algorithms and the state-of-the-art methods on the independent data set

SHAP interaction values are a generalization of SHAP values to higher order interactions. Using SHAP interaction values, we can decompose the impact of a feature on a specific sample into a main effect and interaction effects with other features. Fast exact computation of pairwise interactions are implemented for tree models. This returns a matrix for every prediction, where the main effects are on the diagonal and the interaction effects are off-diagonal. These values often reveal interesting hidden relationships (Lundberg et al., 2020). Below we give some SHAP dependence plot for the features we used, revealing some interesting and unknown trends:

**
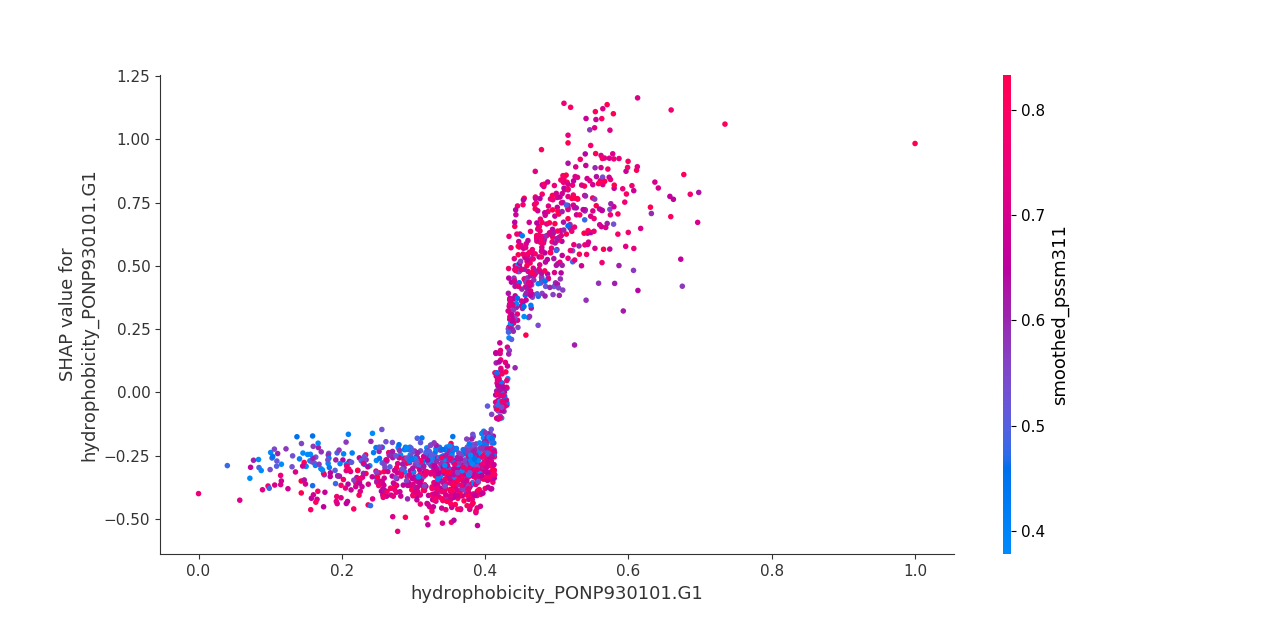
**

**Figure S2.** SHAP dependence plot of hydrophobicity_PONP9-30101.G1 vs. its SHAP value in the T4SE-XGB model


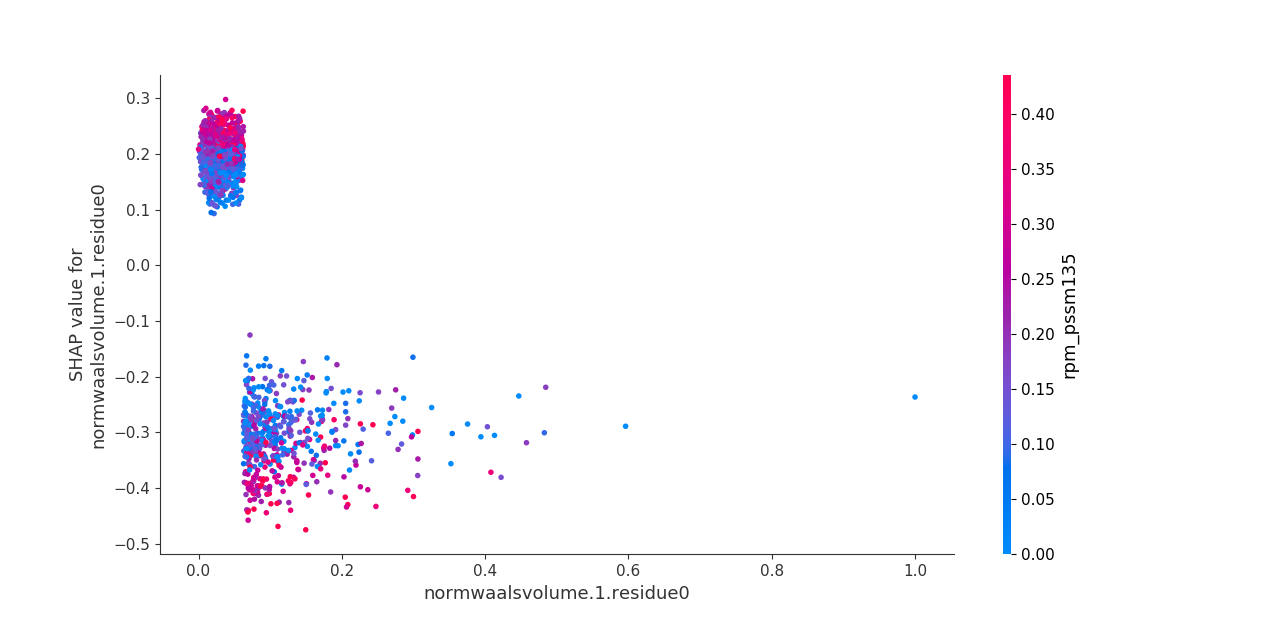


**Figure S3.** SHAP dependence plot of normwaalsvolume.1.residue0 vs. its SHAP value in the T4SE-XGB model

**
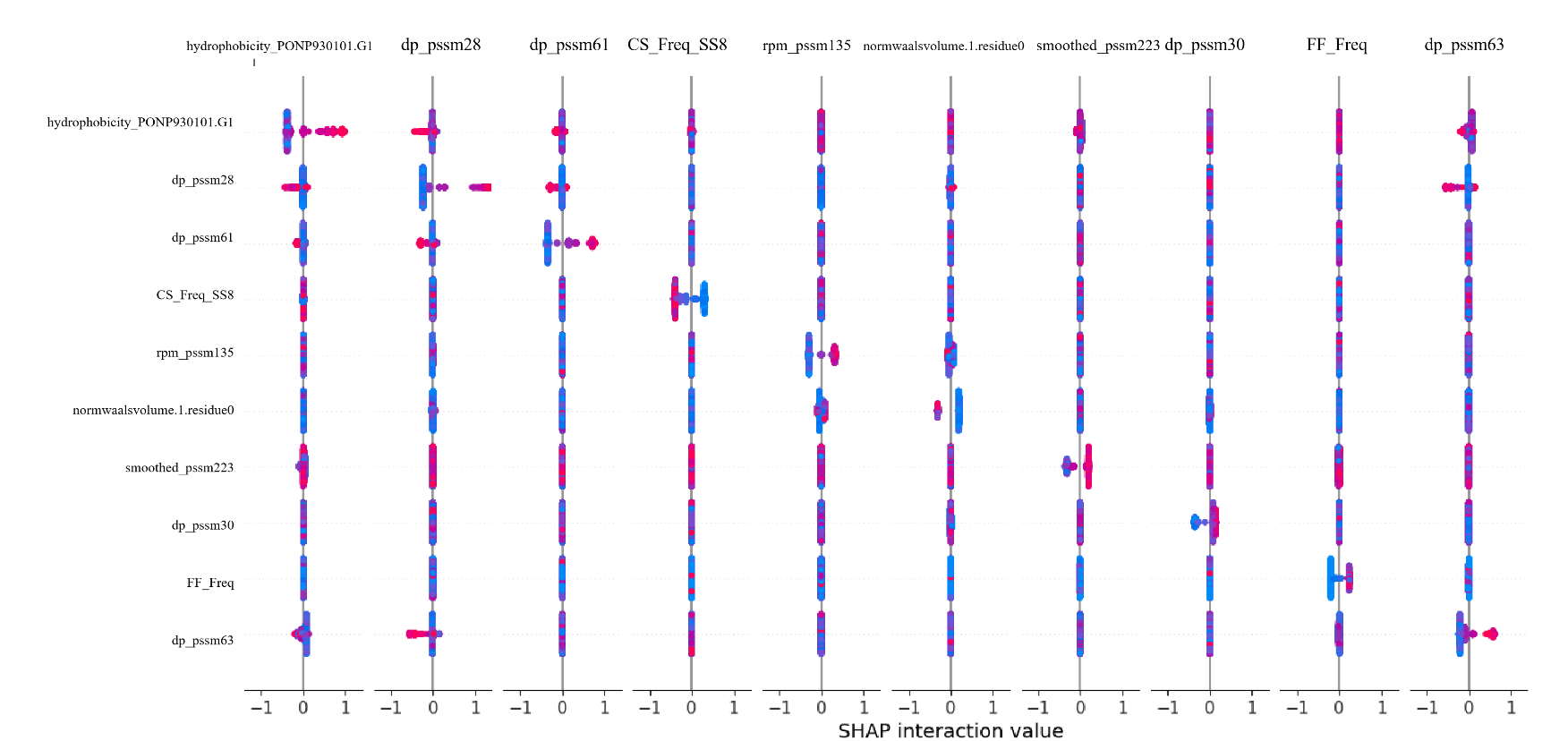
**

**Figure S4.** SHAP Interaction Value Summary Plot: A summary plot of a SHAP interaction value matrix involved the top 10 features plots a matrix of summary plots with the main effects on the diagonal and the interaction effects off the diagonal.

# References

Lundberg, S.M., Erion, G., Chen, H., DeGrave, A., Prutkin, J.M., Nair, B., et al. (2020). From local explanations to global understanding with explainable AI for trees. *Nature Machine Intelligence* 2(1)**,** 56-67. doi: 10.1038/s42256-019-0138-9.
